# Syndrome de Ballantyne associée à un rhabdomyome cardiaque fœtal: à propos d´un cas

**DOI:** 10.11604/pamj.2021.39.116.29610

**Published:** 2021-06-10

**Authors:** Kamal El Moussaoui, Othmane El Harmouchi, Aziz Baidada, Aicha Kharbach

**Affiliations:** 1 Département de Gynécologie Obstétrique et Endoscopie Gynécologique, Maternité Souissi, Centre Hospitalier Universitaire Ibn Sina, Rabat, Maroc

**Keywords:** Syndrome de Ballantyne, syndrome Miroir, hydrops, à propos d´un cas, Ballantyne syndrome, mirror syndrome, hydrops, case report

## Abstract

Le syndrome de Ballantyne ou syndrome en miroir qui a été décrit pour la première fois en 1892 est une pathologie maternelle désignant un syndrome d´anasarque fœtale compliqué d´œdèmes maternels plus ou moins généralisés accompagnés d´albuminurie et parfois d´une anémie. C´est une entité clinique rare. Le diagnostic repose sur une triade qui consiste dans la coexistence d´hydrops fœtal, œdème maternel généralisé et placentomégalie. Il peut être en relation avec l´hydrops fœtal de n´importe quelle cause. Son diagnostic doit être évoqué devant un syndrome œdémateux maternel associé à un état d´anasarque fœtale. Le pronostic fœtal réservé auquel peut s'associer une forte morbidité maternelle expliquent l'intérêt de poser un diagnostic précoce en identifiant son étiologie afin d'établir un traitement anténatal pouvant améliorer ainsi le pronostic materno-fœtal. Nous rapportons un cas unique, jamais décris dans la littérature, d´un syndrome de Ballantyne chez une patiente de 32 ans dont l´étiologie de l´hydrops fœtale était une tumeur cardiaque fœtale type rhabdomyome cardiaque.

## Abstract (en)

Ballantyne syndrome or mirror syndrome was first described in 1892. It is a disorder affecting pregnant women describing the association of fetal anasarca complicated by more or less generalized maternal edema and albuminuria (and sometimes anemia). It is a rare clinical entity. Diagnosis is based on a triad consisting of fetal hydrops, generalized maternal edema and placentomegaly. It can be associated with fetal hydrops from any cause. Diagnostic should be suspected in patients with maternal edema syndrome associated with fetal anasarca. Guarded fetal prognosis can be associated with high maternal morbidity; hence the need for early diagnosis, resting on a clear determination of its cause, and aimed to implement antenatal treatment improving maternal and fetal prognosis. We here report a unique case of Ballantyne syndrome which has never been described in the literature. The study involved a 32-year-old female patient with fetal hydrops caused by fetal cardiac rhabdomyoma.

## Introduction

Le syndrome de Ballantyne a été décrit pour la première fois en 1892 par John W. Ballantyne en 1892, il a également été appelé syndrome du triple œdème, également appelé syndrome du triple œdème ou syndrome miroir, il se caractérise par le développement d'un œdème maternel en association avec une hydrops fœtale. Les piliers fondamentaux du diagnostic sont hydrops fœtal, placentomégalie et l´œdème maternel, il peut être associé aussi à une protéinurie, hypertension artérielle modérée et anémie, ce dernier signe la différencie de la prééclampsie classique.

Au dix-neuvième siècle et jusqu'au milieu du vingtième siècle, elle était associée principalement à un hydrops immunitaire dû à une sensibilisation au rhésus (Rh), pour l'instant il a été décrit en association avec: la maladie d'Ebstein, le chorio-angiome placentaire, tachycardie supra-ventriculaire fœtale, infection à Parvovirus B19, infection à cytomégalovirus, anévrisme de la veine de Galen, fœtus a cardiaque et tératome sacral coccygien [1, 2].

Elle peut survenir au cours du deuxième et du troisième trimestre de la grossesse, elle présente une morbidité périnatale très élevée, la maladie se résout par un accouchement prématuré ou la mort du fœtus. Des cas isolés de résolution par correction de la cause de la mort fœtale. La résolution par la correction de la cause de l'hydrops fœtal a été décrite [3]. Il existe peu de cas publiés, moins d'une centaine, dans la littérature.

Nous avons pu observer dans notre service un cas de pseudo pré-éclampsie en rapport avec une anasarque fœtale grave. La pathologie maternelle était probablement directement liée à la pathologie fœto-placentaire. Nous rapportons un cas rare, d´étiologie jamais décrite par avant dans la littérature ce cas de syndrome de Ballantyne liés à une tumeur cardiaque à type de rhabdomyome.

## Patient et observation

**Figure 1:**
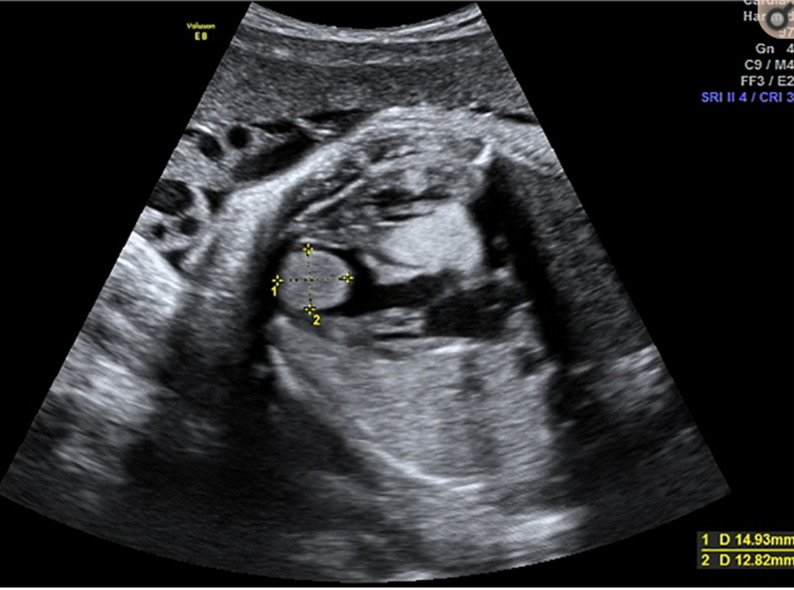
image échographique montrant une masse cardiaque échogène de 14x12mm correspondant un rhabdomyome cardiaque

**Information de la patiente:** il s´agit d´une patiente âgée de 34 ans, sans antécédents médicaux ou chirurgicaux. Son groupe sanguin est O+ et la patiente n´avait aucun antécédent de prise de toxique. Sur le plan obstétrical la patiente était un deuxième geste, deuxième pare. La première grossesse en 2017 menée à terme sans complication, accouchée par voie d´un nouveau-né de sexe féminin de 3400g avec un bon développement psychomoteur. Durant la deuxième grossesse, la patiente a bénéficié de deux échographies en 9 semaines d´aménorrhées (SA) et 12 SA, sans particularité. Les sérologies toxoplasmose, rubéole et VIH étaient négative, un hémogramme normal et des chiffres tensionnelles autour de 120/80mmhg. La patiente a été adressée par son médecin traitant après la découverte à l´échographie du deuxième trimestre d´un début d´anasarque fœtal associée à une image hyperéchogène cardiaque (Figure 1).

**Résultats cliniques:** la patiente est vue en consultation a 28 SA, la patiente était stable sur le plan hémodynamique avec une tension artérielle a 130/80 mm hg, labstix négative, pas de signes neurosensorielles. La patiente a bénéficié d´une échographie chez un référant montrant une grossesse évolutive avec des mensurations correspondant à 28 SA, et des signes de début d´hydrops fœtale avec une ascite de moyenne abondance, associée à un épanchement pleurale minime et un épaississement cutanée fœtale. Au niveau cardiaque l´échographie a montré une masse échogène de 14*12mm au niveau du ventricule gauche ayant l´aspect d´un rhabdomyome cardiaque. Le placenta légèrement épaissie et l´indice de liquide amniotique (IMA) était normal. Les doppler ombilicale et cérébral étaient dans la limite de la normale. Aucune autre anomalie fœtale n'a été détectée, et la vitesse systolique de pointe de l'artère cérébrale moyenne était normale.

**Figure 2:**
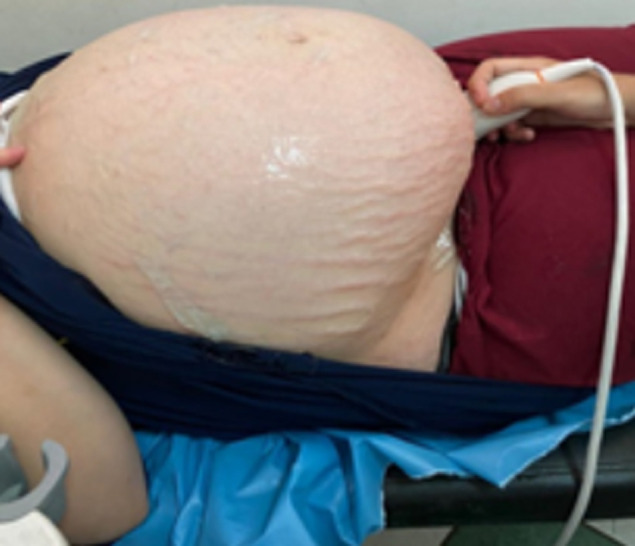
image montrant un abdomen très distendu et la peau abdominales très infiltrées et oedématiées

**Figure 3:**
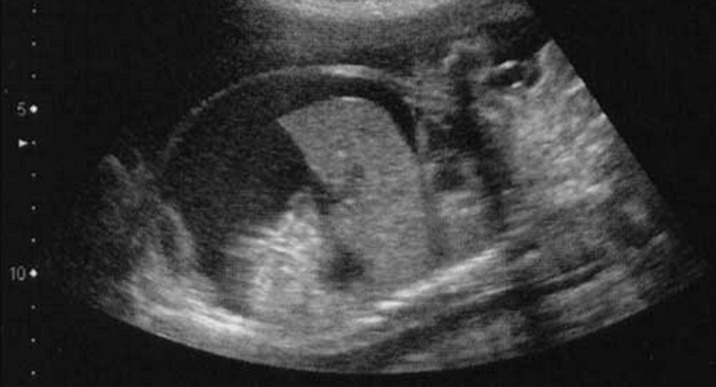
image échographique montrant l'hydrops foetal

**Démarche diagnostique:** biologiquement, la recherche des agglutinines irrégulières est négative, l'hémoglobinémie est à 12,9g/dL, l'hématocrite à 39,7%, les plaquettes à 255000 éléments/mm^3^ et la glycémie à 0,75g/L. La sérologie toxoplasmose, rubéole, VIH, syphilis, CMV étaient négatives. Une maturation fœtale a été administré et la patiente a été suivie ensuite chaque semaine avec des échographies de contrôle, des enregistrements de rythmes cardiaque fœtale; l´évaluation du bien être fœtale et examen maternel. La patiente a été vue pour la dernière fois à la 31^e^SA puis elle ne s´est plus présentée à l´hôpital. A 33 SA, la patiente s´est représenté aux urgences obstétricales dans un tableau d´œdème généralisé avec notion de prise de poids excessifs estimé à 10 kg dans les 5 derniers jours, la tension artérielle était à 140/90, il n´existait pas de signes fonctionnels d´hypertension artérielle, une albuminurie à 2 croix. L´examen clinique retrouve des œdèmes importants au niveau des membres inférieurs, avec un abdomen très distendu et la peau abdominale très infiltrées et œdématiées (Figure 2). Le bilan biologique ne retrouvait pas de cytolyse hépatique, une hémoglobine à 9,1g/dL, un hématocrite à 24,8%, une protidémie à 50g/L et des plaquettes normales. La recherche des agglutinines irrégulières est négative L´échographie obstétricale objectivant une aggravation de l´anasarque fœto-placentaire avec un important hydrops fœtale, avec une ascite de grande abondance, un hydrothorax, un épanchement péricardique, et un important œdème sous cutanée fœtale, et un placenta augmenté de volume (12cm de diamètre). Avec au niveau du cœur, la persistance de cette masse échogène de 40*32mm au niveau du ventricule gauche ayant l´aspect d´un rhabdomyome cardiaque et par ailleurs des signes d´insuffisances cardiaques ont été objectivé avec un doppler ombilical et cérébrales étaient pathologiques; une vitesse systolique cérébrale à 1,4 MoM, et l´enregistrement du rythme cardio fœtale objectivant une souffrance fœtale (Figure 3).

**Figure 4:**
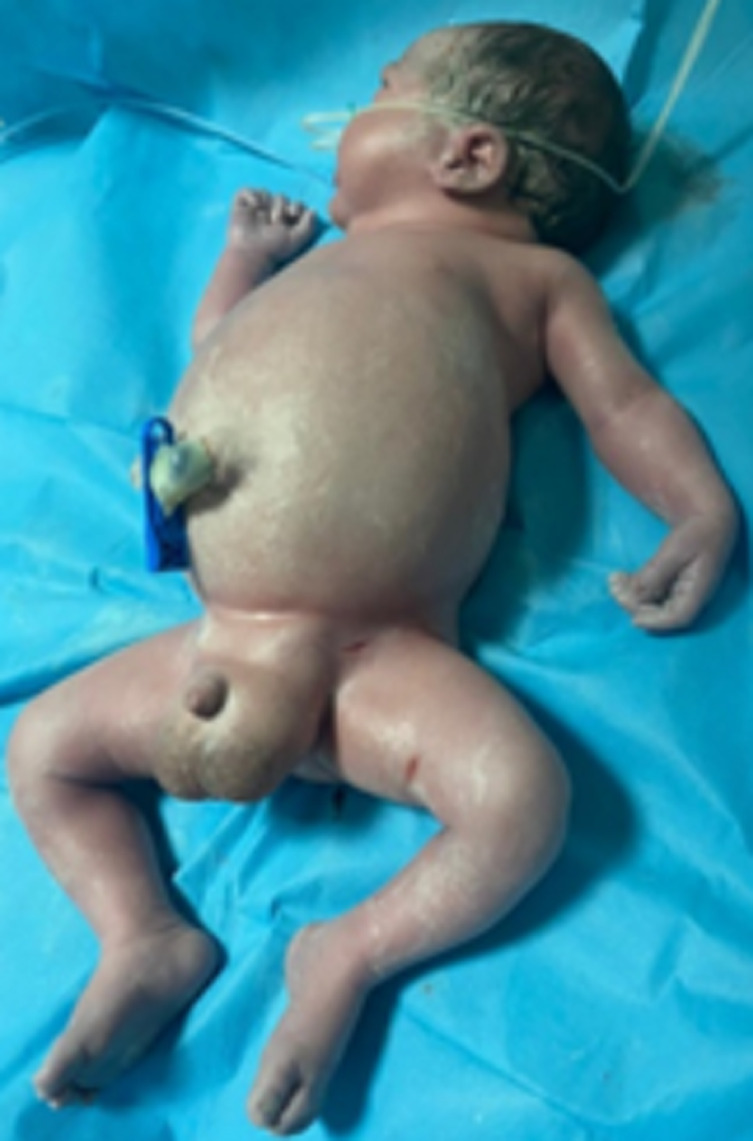
image du nouveau-né à la naissance en anasarque

**Intervention thérapeutique et suivi:** devant l´état d´œdème généralisée d´installation aigüe associée à l´hydrops fœtal et l´hypertrophie placentaire le diagnostic du syndrome miroir a été posé. Devant la souffrance fœtale, Une césarienne a été décidée en urgence pour extraction d´un fœtus de sexe masculin pesant 2950g avec un placenta de 865g très œdématiée, sans contexte d´hémorragie de la délivrance. Le fœtus était en anasarque (Figure 4). Il décèdera à j2 de vie en réanimation pédiatrique. L´examen anatomopathologique objective un placenta hypertrophique avec aspect hydropique. La post opératoire a été marquée par une nette amélioration clinique et biologique et la disparition progressive des œdèmes généralisées maternels avec une disparition complète à J7 du post-partum.

## Discussion

Le syndrome miroir est une complication rare et gravissime de l´hydrops fœtal caractérisé par un œdème maternel « en miroir » de l´anasarque fœtale. Cette entité clinique fut rapportée pour la première fois en 1892 sous le terme de syndrome de Ballantyne, en référence à son auteur, décrivant un tableau maternel pseudo-toxémique secondaire à une anasarque fœto-placentaire d´origine immune par allo-immunisation rhésus [4]. Cette entité clinique portera différents noms au travers des différentes publications qui lui furent consacrées: syndrome de Ballantyne ou syndrome miroir [5]. L´âge gestationnel moyen du diagnostic varie entre 16 et 34 SA [6]. Dans notre observation, le diagnostic fut posé à 28 SA. Il repose sur l´association pathognomonique de trois signes: une anasarque fœtale qu´il soit d´origine immune ou non, un œdème placentaire, un tableau maternel d´œdème avec hémodilution, pouvant aboutir à un état de prééclampsie [7, 8]. L´œdème généralisé et la prise de poids rapide, principaux motifs de consultation chez notre patiente, sont les symptômes communs retrouvés dans 89,3% des cas publiés [6]. D´autres signes cliniques ou biologiques sont retrouvés de manière inconstante: une élévation de la pression artérielle [8, 9]. Des céphalées [10], des troubles de la vision [11], une oligurie, une anémie de dilution, une hypoprotéinémie avec protéinurie [7, 9], une uricémie élevée [12], une élévation des transaminases [13]. Sur le plan biologique, le signe principal est l´hémodilution qui contraste avec l´hémoconcentration observée classiquement en cas de pré-éclampsie [6]. Cette hémodilution s´est manifestée chez notre patiente par un hématocrite, une anémie par hémodilution. Selon Carbillon *et al*. l´anémie de dilution non hémolytique est due à une augmentation du volume sanguin maternel et à une augmentation des concentrations de vasopressine et de l´antidiuretic hormone (ADH) [8].

Le pronostic fœtal du syndrome miroir est très souvent réservé, avec de fréquents décès in utero en rapport avec l´étiologie de l´anasarque. Cependant, c´est le retentissement potentiellement morbide maternel qui en fait la particularité, car il peut aller jusqu´au décès maternel par insuffisance respiratoire (OPA) [14] voir une insuffisance rénale [15]. La particularité du syndrome miroir réside également dans la réversibilité des symptômes maternels après résolution de l´anasarque fœto-placentaire. Cette résolution est généralement obtenue suite à une interruption médicale de grossesse ou, rarement, et quand l´étiologie de l´hydrops le permet, au traitement de celui-ci. Ainsi Ville *et al*.et Duthie *et al*. ont décrit 2 cas de syndrome miroir dû à une infection à parvovirus et dont la correction de l´anémie par transfusion in utero a permis une amélioration de la symptomatologie maternelle et la naissance du nouveau-né à terme en bonne santé [10, 12]. La normalisation d´une arythmie fœtale par administration de flécaïnide a permis une amélioration de la symptomatologie fœtale et maternelle chez une patiente atteinte d´un syndrome miroir [16].

La physio-pathogénie de ce syndrome reste encore incomprise malgré les similitudes cliniques avec la pré-éclampsie. Les mécanismes placentaires semblent différer, l´ischémie placentaire (par défaut d´invasion trophoblastique) serait primitive pour la pré-éclampsie alors qu´elle serait secondaire à une hypovascularisation causée par l´œdème placentaire dans le syndrome miroir. Ceci est confirmé par l´examen anatomopathologique du placenta et contraste avec l´ischémie placentaire et les infarcissements habituels de la pré-éclampsie. L´examen du placenta, dans le cas que nous rapportons décrit cette hyperplacentose avec un placenta d´aspect hydropique. Il n´est donc pas étonnant de retrouver chez les patientes présentant un syndrome miroir une élévation de facteurs anti-angiogéniques tels que le soluble *Vascular Endothelial Growth Factor Receptor-1 (sVEGFR-1)* et le soluble *Fms-like tyrosine kinase (sFlt1)* et une diminution de facteurs angiogéniques tels que le *Placental Growth Factor (PIGF)* [17]. Cette ischémie placentaire et ce dérèglement endothélial sont à l´origine de libération de radicaux libres, lipides oxydés, cytokines et leptines responsables d´une augmentation de la perméabilité vasculaire, d´une vasoconstriction artérielle et d´une activation des systèmes de coagulation intravasculaire maternels qui expliqueraient les manifestations cliniques et biologiques du syndrome miroir [18].

Dans notre cas, et pour la première fois dans la littérature, l´étiologie de l´anasarque fœtale responsable de syndrome miroir est le rhabdomyome cardiaque. Les rhabdomyomes cardiaques fœtaux sont des tumeurs cardiaques rares, l'incidence des rhabdomyomes cardiaques fœtaux dans la phase postnatale est d'environ 1/40000. L'étiologie est inconnue, mais certaines données indiquent que les hormones maternelles sont impliquées [19]. Grâce à l'amélioration de la technologie échographique et à une plus grande expérience, les rhabdomyomes cardiaques fœtaux sont maintenant plus fréquemment diagnostiqués en phase prénatale. Ils se présentent comme des masses hyperéchogènes homogènes non vasculaires provenant du myocarde. Ils peuvent être localisés dans toutes les zones du myocarde mais sont généralement détectés dans le septum ou les ventricules. Le diagnostic est souvent fortuit.

Les rhabdomyomes cardiaques peuvent continuer à se développer dans le post-partum, en raison des œstrogènes maternels. La diminution ultérieure de la taille de la tumeur peut être due à la réduction du taux d'œstrogènes [20]. Ces tumeurs sont les plus grosses dans la vie fœtale, puis diminuent avec l'âge et peuvent même disparaître complètement. La prévalence est donc plus élevée chez les enfants que chez les adultes [21]. Les rhabdomyomes cardiaques régressent généralement sans traitement, mais une résection chirurgicale doit être effectuée si la fonction cardiaque est affectée. Le pronostic est bon s'il n'y a pas de complication in utero et ou dans les six premiers mois postnatals, mais il est de mauvais pronostique en cas de dysfonctionnement cardiaque. Le taux de mortalité néonatale est de 4 - 6% [20].

## Conclusion

L´anasarque fœtale, quelle qu´en soit la cause, lorsqu´il est associé à un syndrome maternel pré-éclamptique, doit faire évoquer et rechercher le syndrome de Ballantyne, dont on connaît le pronostic fœtal très péjoratif, et maternel potentiellement grave. On sait maintenant que la résolution de la pathologie fœtale peut entraîner l´amélioration de la symptomatologie maternelle et autoriser éventuellement la poursuite de la grossesse.
